# Efficacy and Safety of Elamipretide in Individuals With Primary Mitochondrial Myopathy: The MMPOWER-3 Randomized Clinical Trial

**DOI:** 10.1212/WNL.0000000000207402

**Published:** 2023-07-18

**Authors:** Amel Karaa, Enrico Bertini, Valerio Carelli, Bruce H. Cohen, Gregory M. Enns, Marni J. Falk, Amy Goldstein, Gráinne Siobhan Gorman, Richard Haas, Michio Hirano, Thomas Klopstock, Mary Kay Koenig, Cornelia Kornblum, Costanza Lamperti, Anna Lehman, Nicola Longo, Maria Judit Molnar, Sumit Parikh, Han Phan, Robert D.S. Pitceathly, Russell Saneto, Fernando Scaglia, Serenella Servidei, Mark Tarnopolsky, Antonio Toscano, Johan L.K. Van Hove, John Vissing, Jerry Vockley, Jeffrey S. Finman, David A. Brown, James A. Shiffer, Michelango Mancuso, for the MMPOWER-3 Trial Investigators

**Affiliations:** From the Massachusetts General Hospital (A.K.), Harvard Medical School Boston; Neuromuscular Unit (E.B.), Bambino Gesù Ospedale Pediatrico, IRCCS, Rome; IRCCS Istituto delle Scienze Neurologiche di Bologna (V.C.), Programma di Neurogenetica; Department of Biomedical and Neuromotor Sciences (V.C.), University of Bologna, Italy; Rebecca D. Considine Research Institute (B.H.C.), Akron Children's Hospital, OH; Stanford University School of Medicine (G.M.E.), CA; Mitochondrial Medicine Frontier Program (M.J.F., A.G.), Division of Human Genetics, Department of Pediatrics, Children's Hospital of Philadelphia and University of Pennsylvania Perelman School of Medicine; Royal Victoria Infirmary (G.S.G.), Newcastle upon Tyne, United Kingdom; University of California (R.H.), San Diego, La Jolla; Columbia University Irving Medical Center (M.H.), New York; Friedrich-Baur-Institute (T.K.), Department of Neurology, LMU Hospital, Ludwig Maximilian University of Munich; German Center for Neurodegenerative Diseases (DZNE); Munich Cluster for Systems Neurology (SyNergy), Germany; Department of Pediatrics (M.K.K.), University of Texas McGovern Medical School, Houston; Department of Neurology, Neuromuscular Diseases Section (C.K.), University Hospital of Bonn, Germany; Fondazione IRCCS Istituto Neurologico Carlo Besta (C.L.), Milano, Italy; Vancouver General Hospital (A.L.), British Columbia, Canada; University of Utah (N.L.), Salt Lake City; Institute of Genomic Medicine and Rare Disorders (M.J.M.), Semmelweis University, Budapest, Hungary; Cleveland Clinic Neurological Institute (S.P.), OH; Rare Disease Research (H.P.), Atlanta, GA; Department of Neuromuscular Diseases (R.D.S.P.), UCL Queen Square Institute of Neurology and The National Hospital for Neurology and Neurosurgery, London, United Kingdom; Seattle Children's Hospital (R.S.), WA; Baylor College of Medicine (F.S.), Houston, TX; Texas Children's Hospital (F.S.); Joint BCM-CUHK Center of Medical Genetics (F.S.), Hong Kong SAR; Fondazione Policlinico Universitario A. Gemelli and Istituto di Neurologia (S.S.), Università Cattolica del Sacro Cuore, Rome, Italy; McMaster University Medical Center (M.T.), Hamilton, Ontario, Canada; Neurology and Neuromuscular Unit (A.T.), Department of Clinical and Experimental Medicine, University of Messina, Italy; University of Colorado and Children's Hospital Colorado (J.L.K.V.H.), Aurora; Copenhagen Neuromuscular Center (John Vissing), Rigshospitalet University of Copenhagen, Denmark; Children's Hospital of Pittsburgh (Jerry Vockley), University of Pittsburgh School of Medicine, PA; Jupiter Point Pharma Consulting (J.S.F.), LLC; Stealth BioTherapeutics (D.A.B.)Write On Time Medical Communications (J.A.S.), LLC; and Department of Clinical and Experimental Medicine (M.M.), Neurological Institute, University of Pisa, Italy.

## Abstract

**Background and Objectives:**

Primary mitochondrial myopathies (PMMs) encompass a group of genetic disorders that impair mitochondrial oxidative phosphorylation, adversely affecting physical function, exercise capacity, and quality of life (QoL). Current PMM standards of care address symptoms, with limited clinical impact, constituting a significant therapeutic unmet need. We present data from MMPOWER-3, a pivotal, phase-3, randomized, double-blind, placebo-controlled clinical trial that evaluated the efficacy and safety of elamipretide in participants with genetically confirmed PMM.

**Methods:**

After screening, eligible participants were randomized 1:1 to receive either 24 weeks of elamipretide at a dose of 40 mg/d or placebo subcutaneously. Primary efficacy endpoints included change from baseline to week 24 on the distance walked on the 6-minute walk test (6MWT) and total fatigue on the Primary Mitochondrial Myopathy Symptom Assessment (PMMSA). Secondary endpoints included most bothersome symptom score on the PMMSA, NeuroQoL Fatigue Short-Form scores, and the patient global impression and clinician global impression of PMM symptoms.

**Results:**

Participants (N = 218) were randomized (n = 109 elamipretide; n = 109 placebo). The m0ean age was 45.6 years (64% women; 94% White). Most of the participants (n = 162 [74%]) had mitochondrial DNA (mtDNA) alteration, with the remainder having nuclear DNA (nDNA) defects. At screening, the most frequent bothersome PMM symptom on the PMMSA was tiredness during activities (28.9%). At baseline, the mean distance walked on the 6MWT was 336.7 ± 81.2 meters, the mean score for total fatigue on the PMMSA was 10.6 ± 2.5, and the mean T score for the Neuro-QoL Fatigue Short-Form was 54.7 ± 7.5. The study did not meet its primary endpoints assessing changes in the 6MWT and PMMSA total fatigue score (TFS). Between the participants receiving elamipretide and those receiving placebo, the difference in the least squares mean (SE) from baseline to week 24 on distance walked on the 6MWT was −3.2 (95% CI −18.7 to 12.3; *p* = 0.69) meters, and on the PMMSA, the total fatigue score was −0.07 (95% CI −0.10 to 0.26; *p* = 0.37). Elamipretide treatment was well-tolerated with most adverse events being mild to moderate in severity.

**Discussion:**

Subcutaneous elamipretide treatment did not improve outcomes in the 6MWT and PMMSA TFS in patients with PMM. However, this phase-3 study demonstrated that subcutaneous elamipretide is well-tolerated.

**Trial Registration Information:**

Trial registered with clinicaltrials.gov, Clinical Trials Identifier: NCT03323749; submitted on October 12, 2017; first patient enrolled October 9, 2017. clinicaltrials.gov/ct2/show/NCT03323749?term = elamipretide&draw = 2&rank = 9.

**Classification of Evidence:**

This study provides Class I evidence that elamipretide does not improve the 6MWT or fatigue at 24 weeks compared with placebo in patients with primary mitochondrial myopathy.

A consensus of experts define primary mitochondrial myopathies (PMMs) as a diverse group of genetically confirmed disorders of the mitochondria, affecting predominantly, but not exclusively, skeletal muscle, thereby adversely affecting physical function and quality of life.^1,2^ The result is muscle weakness, muscle atrophy, limited exercise capacity, and symptoms of fatigue and pain.^1,2^ PMM severity is variable, but the progressive reduction in exercise capacity eventually impairs participants' ability to perform activities of daily living.^3-5^

Primary mitochondrial diseases (PMDs) caused by both mitochondrial (mtDNA) and nuclear DNA (nDNA) alteration are among the most common inherited metabolic disorders.^2^ PMDs have been reported to affect at least 1 in 4,300 people in the general population^6^ or an estimated 40,000 total individuals in the United States.^6^ Because most patients with PMDs are reported to experience PMM, the prevalence of PMM specifically is estimated to be slightly less than the overall prevalence of all PMDs (aside from patients with Leber hereditary optic neuropathy [LHON]) who do not experience a skeletal muscle component).^6,7^

Currently, available standards of care primarily use dietary supplements that have limited clinical impact.^8^ Therefore, a significant unmet clinical need for new therapies exists.^8^ However, there have been a number of historical challenges to the development of mitochondrial therapies, including the lack of a specific molecular target in mitochondria to promote adenosine triphosphate synthesis and a drug development process that is driven by disease-specific approaches.^9^

Elamipretide is an investigational mitochondrial-targeting agent in development for treating patients with a variety of mitochondrial diseases.^10-13^ Elamipretide is a water-soluble, aromatic, cationic, mitochondria-targeting tetrapeptide that readily penetrates and transiently localizes to the inner mitochondrial membrane where it associates with cardiolipin to improve membrane stability and restore supercomplex formation, thereby enhancing adenosine triphosphate synthesis in several organs including the heart, kidney, neurons, and skeletal muscle, and reduce reactive oxygen species (ROS) production.^10,13-26^ High-resolution respirometry studies in human and animal models of myopathy have demonstrated elamipretide-mediated improvement of respiration across various electron transport chain complexes.^27,28^ These effects corresponded with significantly improved mitochondrial and cristae morphology,^27^ which are known to be altered across many mitochondrial myopathies.^29^

The elamipretide clinical development program included MMPOWER-1 and MMPOWER-2, whereby treatment with elamipretide demonstrated meaningful improvements in patient-reported outcomes (PROs) for patients with confirmed PMM.^10,11^ MMPOWER-3 was a pivotal, phase 3, randomized, double-blind, placebo-controlled clinical trial designed to evaluate the efficacy and safety of 40 mg of elamipretide subcutaneously (SC) once daily for 24 weeks as a treatment for patients with PMM using the 6-minute walk test (6MWT) and fatigue questionnaires as outcome measures. MMPOWER-3 was designed to provide important baseline characteristics and data on how treatment may affect functional changes and PROs.

## Methods

### Study Design and Participants

MMPOWER-3 was a 24-week , randomized, double-blind, parallel-group, placebo-controlled clinical trial for adults with PMM conducted at 27 clinical research centers in 7 countries (Canada, Denmark, England, Germany, Hungary, Italy, and the United States).

MMPOWER-3 trial participants were primarily identified by the RePOWER registry, a global, prospective, noninterventional registry enrolling 413 ambulatory individuals 16–80 years of age with signs and/or symptoms of PMM.^30^ Registry individuals provided demographic, genetic/phenotypic, functional, and clinical assessments, which were used to confirm genotypic-phenotypic correlations and identify potential phase 3 trial participants before MMPOWER-3 screening.^30^

After screening (7–28 days), eligible participants were randomized in a 1:1 ratio to receive either 24-weeks of once-daily SC dosing of 40 mg of elamipretide or placebo. Study drug or placebo was self-administered subcutaneously by trained participants or their caregivers, at rotating sites around 4 quadrants of the abdomen or the thighs. Treatment began at the baseline visit with assessments at weeks 4, 12, and 24.

Eligible participants were those between 16 years or older and 80 years or younger (18 years or older in Germany), diagnosed with PMM with a confirmed sequence alteration affecting mitochondrial function, and those with symptoms (i.e., exercise intolerance, fatigue, muscle weakness) and/or physical examination findings consistent with a myopathy as the predominant manifestation of their mitochondrial disease. In addition, participants had to be willing and able to provide consent and adhere to trial requirements for inclusion. An acceptable form of birth control was required of participants of childbearing potential during the study.

Participants walking <100 meters or >450 meters during the 6MWT at screening/baseline were also excluded. Participants were not allowed to have had a recent (within 30 days) or planned hospitalization/procedure and were excluded if they had a clinically significant end-organ damage in the opinion of the investigator.^30^

### Standard Protocol Approvals, Registrations, and Patient Consents

MMPOWER-3 was conducted in accordance with international ethics guidelines, including the Declaration of Helsinki, Council for International Organizations of Medical Sciences International Ethical Guidelines, ICH GCP guidelines, and all applicable laws and regulations. The study was approved by institutional review boards, and all participants provided written informed consent (clinicaltrials.gov, Clinical Trials Identifier: NCT03323749.).

### Randomization and Masking

Assignment to treatment groups within each cohort for the randomized portion of the study was determined by a computer-generated random sequence using an Interactive Web-Response System to assign identical glass vials containing either the elamipretide or a placebo, which consisted of the same formulation without elamipretide. Participants were stratified by the subclassification of the specific sequence alteration causing their PMM, as determined by the adjudication committee formed to review and confirm eligibility for study enrollment.^3^ The pharmacists, investigators and trial staff, sponsor, and participants were blinded to treatment.

### Study Assessments and Procedures

MMPOWER-3 was designed to assess the safety and efficacy of elamipretide through primary and secondary clinical study endpoints.

#### Efficacy Assessments

Coprimary endpoints evaluated the effect of elamipretide for 24 weeks including the distance walked (in meters) on the 6MWT and the total fatigue score on the Primary Mitochondrial Myopathy Symptom Assessment (PMMSA), previously described in detail.^31^ The full PMMSA assesses the severity of 10 of the most common symptoms of PMM using the following 4-point scale (not at all^1^ to severe^4^; described later). PMMSA total fatigue score (TFS) focuses on myopathic symptoms most commonly associated with PMM (severity of tiredness and muscle weakness at rest and during activities, as described by participants).

Most bothersome symptom score on the PMMSA and the NeuroQoL Short-Form fatigue scores were secondary endpoints. The Neuro-QoL evaluates and monitors sensations ranging from tiredness to an overwhelming, debilitating, and sustained sense of exhaustion that decreases capacity for physical, functional, social, and mental activities, based on a 5-point scale (1 = never, 2 = rarely, 3 = sometimes, 4 = often, and 5 = always).

Other secondary endpoints included the patient global impression (PGI) and clinician global impression (CGI) of PMM symptoms. PGI and CGI assess patient and clinician overall assessment of the severity of patients' symptoms related to PMM on a 5-point scaled question scored 0 to 4 (0 = none, 1 = mild, 2 = moderate, 3 = severe, and 4 = very Severe) and changes to their symptoms on a 7-point scale scored −3 to 3 (−3 = very much worse, −2 = moderately worse, −1 = a little worse, 0 = no change, 1 = a little better, 2 = moderately better, and 3 = very much better).

The PMMSA was performed during screening, baseline, and daily throughout the 24-week study period. Other efficacy endpoints were performed at screening, baseline, and at weeks 4, 12, and 24 of randomized treatment.

#### Safety Assessments

Safety and tolerability of elamipretide at a dose of 40 mg/d SC were assessed through recording of adverse events (AEs), ascertained through self-report, vital signs, physical examination, ECGs, and clinical laboratory evaluations. Adverse events were assessed for severity and relationship to study medication throughout the 24-week study. Safety measures were assessed during screening, baseline and weeks 4, 12, and 24.

### Statistical Analysis

A sample size of 202 participants, with 101 participants in each treatment arm, was determined to provide 90% power to detect a 30-meter difference between treatment groups in the 6MWT and a 90% power to detect a 1-unit difference in the PMMSA TFS. This was assuming standard deviations of 60 meters for 6MWT and 2 units for the PMMSA TFS, at an alpha level of 0.025, as established from a previous study of elamipretide in patients with PMM.^11^ The 2-sided alpha level of 0.025 was used to account for a possible multiplicity adjustment for the primary efficacy endpoints.

Efficacy was assessed in the intention-to-treat (ITT) population, defined as all participants who received at least 1 dose of investigational medication, and the per-protocol (PP) population, which included all ITT participants without defined protocol violations/deviations identified per blinded data review before database lock. These protocol violations/deviations included not meeting inclusion/exclusion criteria or having a selected major protocol deviation deemed to potentially affect efficacy findings; not completing the study; not receiving investigational treatment within 2 days before the week 24 visit; having <80% compliance to investigational product; and not completing the study. Safety was assessed in the safety population, defined as all participants who received at least 1 dose of investigational medication.

Primary and secondary efficacy outcomes were assessed as the change from baseline to each on-treatment time point with the primary time point being end of treatment (week 24). Analyses of continuous endpoints were conducted using a mixed-model repeated measures approach, with fixed effects for treatment, visit, the treatment-by-visit interaction, and participant as a random effect. The baseline value and a baseline-by-visit interaction for the endpoint were included as covariates. A family-wise alpha level of 0.05 was maintained for the primary endpoints, using Hochberg procedure at the primary time point of 24 weeks. If both primary endpoints were significantly different from placebo at the 0.05 (2-sided ) level of significance in favor of elamipretide, then both endpoints were considered statistically significant. If not, the endpoint with the smaller *p* value of the 2 was considered statistically significant if the *p* value was ≤0.025 (2-sided ).

In the event that both endpoints in the primary endpoint family were significant at the 5% level, then secondary endpoints were to be tested at week 24 with type I error control, achieved by testing sequentially using a 2-sided alpha level of 0.05. The endpoints and hierarchy of comparisons were as follows: (1) change from baseline in Neuro-QoL Fatigue Short-Form (T score); (2) change from baseline in PGI of PMM symptoms; (3) change from baseline in CGI of PMM symptoms; and (4) change from baseline in most bothersome symptom score on the PMMSA. Sequential comparisons to control type I error were only to be completed if previous comparisons were statistically significant. For these analyses, *p* values were nominal.

#### Subgroup Analyses by PMM Genotype

Given the extensive genetic heterogeneity of the study population, an exploratory analysis of genetic subgroups by genomic alteration (mtDNA and nDNA) was performed using the same methods as described for the primary efficacy endpoints earlier (ITT population using similar mixed-model repeated measure [MMRM] models).

Because of a potential data entry error, which was later identified in post hoc data analysis, 3 participants were misclassified in the clinical study report as having a pathogenic mtDNA variant. Post hoc analyses revealed that these 3 participants instead had nDNA alteration, either in *POLG* (2 participants) or *TWNK* (in 1 participant). Accordingly, these participants were moved from the mtDNA group into the nDNA cohort for the included genetic alteration analyses. 6MWT for 1 participant at week 24 was deemed unusable because the participant inadvertently received walking assistance and was therefore not included in the 6MWT analyses. Study protocol and statistical analysis plan were published on ClinicalTrials.gov updated on January 24th 2022 (NCT03323749).^32^

#### Pharmacokinetic Analysis

The PK population included 106 participants randomized and treated with elamipretide, with at least 1 PK sample taken during their participation. PK modeling for elamipretide and its metabolites, M1 and M2, were performed using NONMEM computer software. Covariates, such as age, genotype, weight, height, lean body mass, body mass index, liver function tests, serum creatinine, and renal function (as described by estimated glomerular filtration rate [eGFR]) were analyzed. The exposure-response analysis examined response based on the 6MWT as a function of steady-state exposure to elamipretide and its metabolites.

### Role of the Funding Source

The funding source for this study participated in the development of the study design. All authors participated in data collection, data interpretation, and the clinical study report writing. The manuscript lead author had full access to the totality of the study data. The remaining authors were provided with an aggregate data analysis. All authors had final responsibility for the decision to submit for publication.

### Data Availability

Anonymized data not published within this article will be made available by request from any qualified investigator.

### Protocol and Statistical Analysis Plan

The study protocol and statistical analysis plan were published.^32^

## Results

### Participants

**Figure 1:**
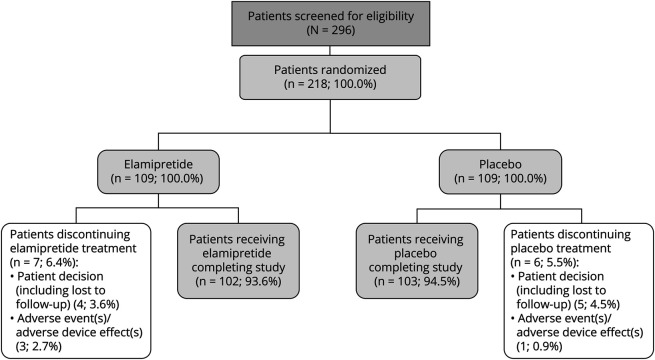
Participant Disposition Two hundred ninety-six participants were screened, and 218 participants were randomized to treatment. Two participants in the elamipretide group and 1 patient in the placebo group had the treatment withdrawn due to adverse event before study discontinuation. The ITT population included 109 participants in the elamipretide group and 109 participants in the placebo group. The PP population included 102 participants in the elamipretide group and 103 participants in the placebo group. ITT = intention-to-treat; PP = per-protocol.

Of the 296 participants screened for eligibility in MMPOWER-3, 218 were enrolled and randomized to treatment (elamipretide n = 109; placebo n = 109) between October 2017 and December 2019 (Figure 1). Of those receiving investigational product (n = 218), 205 (94%) completed the double-blind period of the study, with a similar percentage of participants for each treatment group completing (n = 102 [93.6%] elamipretide and n = 103 [94.5%] placebo). Thirteen randomized participants (6.0%) discontinued treatment (main reason being participant decision; n = 9 [4.1%]). Most of the participants (90.8%; n = 198/218) were included in the PP patient population (n = 96 [88.1%] for elamipretide and n = 102 [93.6%] for placebo).

Participant demographics at baseline were similar between treatment groups, and characteristics demonstrated similar impairment in PMM (Table 1). Of the 218 treatment-randomized participants, the mean age was 44.9 years with participants mostly being White (94%; n = 203/218) and female (64.2% n = 140/218). The mean weight was 66.0 (±18.9) kg, height was 165.7 (±10.4) cm, and BMI was 24.0 (±6.0) kg/m^2^. Among the participants who completed the 6MWT at baseline, the average distance walked was 330.28 (±76.5) meters. One participant had a protocol violation of walking >450 meters on the 6MWT at baseline. The mean PMMSA TFS was 10.6 (±2.5). The Neuro-QoL Fatigue Short-Form average T score was 55.0 (±7.5) points. At screening, participants reported tiredness during activities (28.9%; n = 63), muscle weakness during activities, (21.1%; n = 46), balance problems (11.5%; n = 25), and tiredness at rest (10.6%; n = 23) on the PMMSA as the most bothersome symptoms of the 10 symptoms of PMM, with bothersome symptoms varying slightly between treatment groups (Table 2).

**Table 1:**
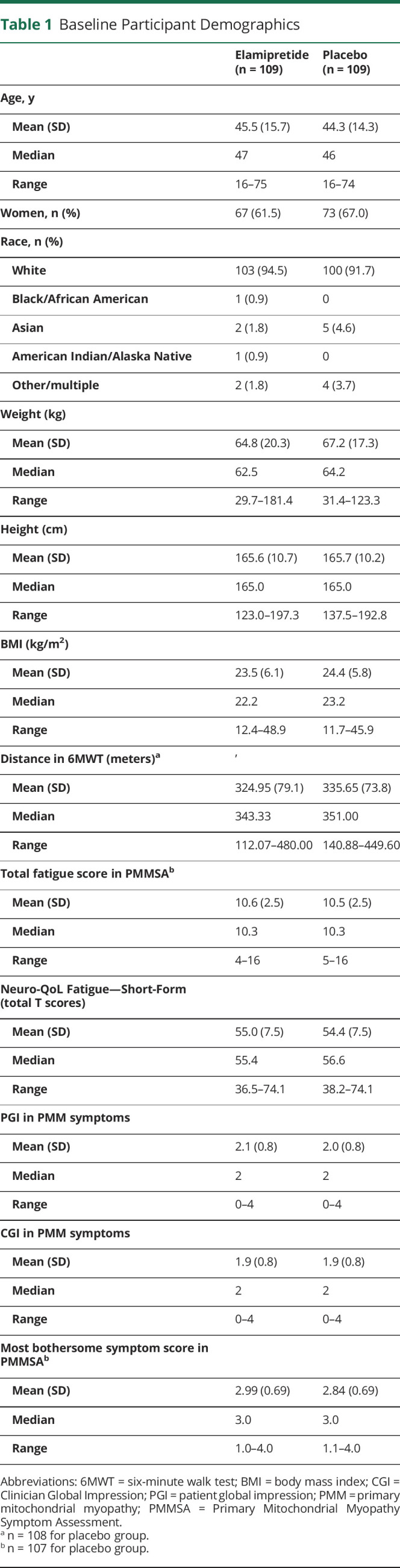
Baseline Participant Demographics

**Table 2:**
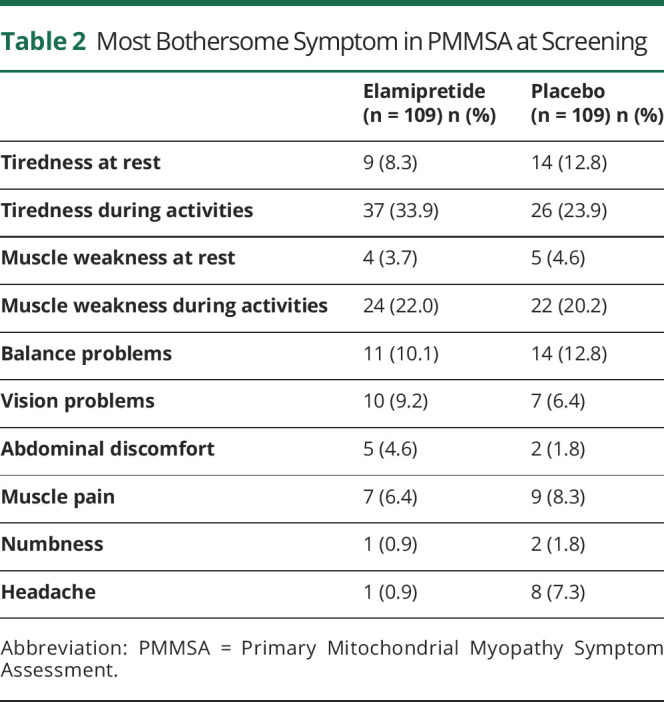
Most Bothersome Symptom in PMMSA at Screening

Baseline genetic test results showed most of the participants (74%, n = 162) had mtDNA alteration, with the remainder (26%, n = 56) having nDNA defects (Table 3 and eFigure 1, links.lww.com/WNL/C851). Because participants were stratified by the subclassification of the genetic class, the distribution of genetic class between mtDNA and nDNA was similar between treatment groups. Three participants were misclassified in the clinical study report (identified in a post hoc analysis) as having a pathogenic mtDNA variant. Instead, these 3 participants had nDNA alteration (either in *POLG* [2 participants] or *TWNK* [in 1 participant]). Accordingly, these participants were moved from the mtDNA group into the nDNA cohort for the genetic alteration analyses, resulting in 73% (n = 159) with mtDNA alteration and the remaining 27% (n = 59) with nDNA defects.

**Table 3:**
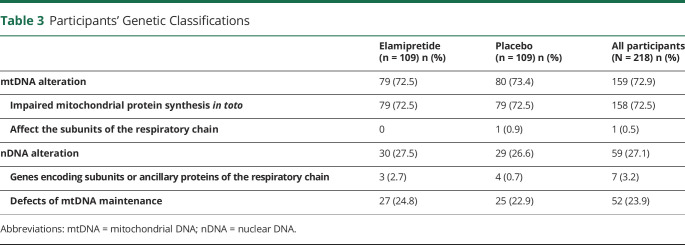
Participants' Genetic Classifications

### Efficacy

#### Primary Endpoints of Overall ITT

**Figure 2:**
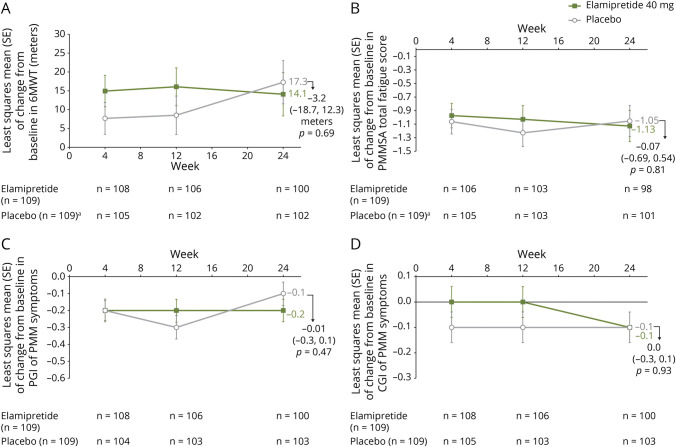
MMPOWER3 Change in Endpoints From Baseline to End of Treatment (Week 24) In 6-minute walk test (6MWT) (A), in Primary Mitochondrial Myopathy Symptom Assessment Total Fatigue Score (PMMSA) (B), in patient global impression (PGI) of Primary Mitochondrial Myopathy Symptoms (C), and in clinician global impression (CGI) of Primary Mitochondrial Myopathy Symptoms (D). ^a^No baseline measurements for 2 participants in the placebo group.

Analysis of the 6MWT at the end of treatment showed the least squares mean (LS) (SE) of change from baseline in distance walked at week 24 was 14.1 (±5.7) meters for participants receiving elamipretide and 17.3 (±5.7) meters for participants receiving placebo, a −3.2-meter difference between the 2 groups (95% CI −18.7 to 12.3; *p* = 0.69) (Figure 2A). The per-protocol (PP) participant analysis demonstrated a −2.2-meter difference between the 2 groups (95% CI −16.9 to 12.5; *p* = 0.77).

Elamipretide-treated participants reported more total fatigue at baseline and less total fatigue at end of treatment, as assessed by the PMMSA TFS. The LS mean (SE) of change from baseline to week 24 on the PMMSA TFS was −1.13 (±0.22) for participants receiving elamipretide and −1.05 (±0.22) for participants receiving placebo, a −0.07 difference between the 2 groups (95% CI −0.69 to 0.54; *p* = 0.81) (Figure 2B). The PP participant analysis demonstrated a 0.09 difference between the 2 groups (95% CI −0.54 to 0.72; *p* = 0.78).

Secondary endpoint results are summarized in eTable 1 (links.lww.com/WNL/C852). Analyses of change from baseline in PGI of PMM symptoms and CGI of PMM symptoms at the end of treatment are provided in Figure 2, C and D, respectively.

#### Analyses by Genetic Subgroup: mtDNA vs nDNA

**Figure 3:**
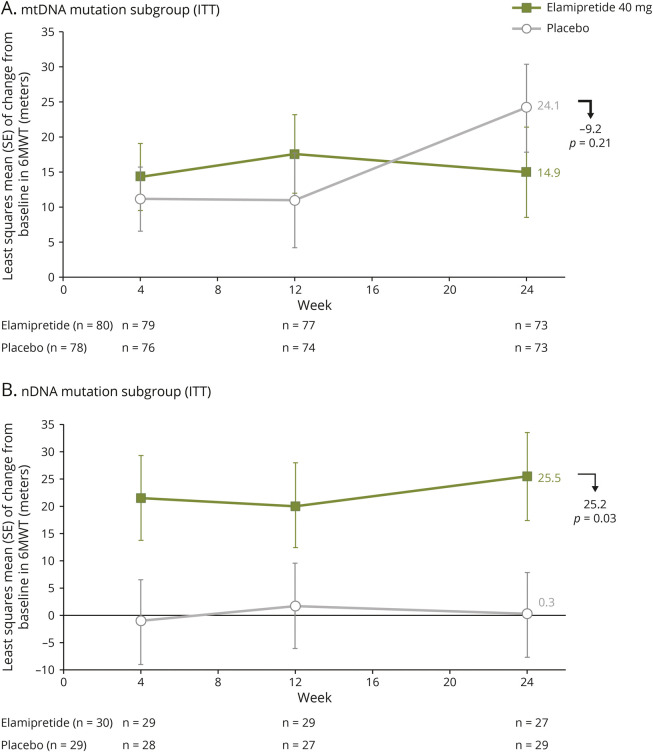
Change From Baseline to End of Treatment (Week 24) in 6-Minute Walk Test: Subgroup Analysis by Genetic Abnormality Subgroup analysis by genetic abnormality for change from baseline to end of treatment (week 24) in 6MWT for (A) participants with mtDNA alteration and (B) participants with nDNA alteration.

mtDNA: Subgroup analysis for participants with mtDNA alteration of the 6MWT at the end of treatment showed the LS mean (SE) of change from baseline in distance walked at week 24 was 14.0 (±6.1) meters for participants receiving elamipretide (n = 74) and 25.0 (±6.1) meters for participants receiving placebo (n = 79), an −11.0-meter between-group difference favoring placebo (95% CI −28.1 to 6.1; *p* = 0.21; Figure 3A).

nDNA: For participants with nDNA alteration (post hoc analysis), the LS mean (SE) change from baseline in distance walked at week 24 was 25.5 (±8.0) meters for participants receiving elamipretide (n = 29) and 0.3 (±7.7) meters for participants receiving placebo (n = 29), a 25.2-meter difference between the 2 groups favoring elamipretide (95% CI 3.1–47.3; *p* = 0.03; Figure 3B).

For participants with mtDNA alteration, the LS mean (SE) of change from baseline at week 24 on the PMMSA TFS was −1.3 (±0.2424) for participants receiving elamipretide and −1.1 (±0.2525) for participants receiving placebo, a −0.21 difference between the 2 groups (95% CI −0.9 to 0.5; *p* = 0.55). For participants with nDNA alteration (post hoc analysis), LS mean (SE) of change from baseline at week 24 was −0.45 (±0.25) for participants receiving elamipretide and −0.48 (±0.24) for participants receiving placebo, a 0.03 difference between the groups (*p* = 0.93).

### Safety

In total, 109 participants received elamipretide and 109 received placebo. AEs during the treatment period were reported by a higher percentage of elamipretide-treated participants (98.2% [n = 107/109]) than placebo-treated participants (76.1% [n = 83/109]) (Table 4). Most AEs in the elamipretide group (97.2%) and half of AEs in the placebo group (51.4%) were reported as treatment-related AEs. Most of the AEs were mild or moderate in intensity. The most commonly reported AEs for participants receiving elamipretide (frequency >10%) were injection site reactions (see Table 4). Injection site reactions experienced with elamipretide included erythema, pruritus, pain, swelling, induration, bruising, hemorrhage, urticaria, and injection site nodules and masses. A low percentage of serious adverse events (SAEs) were reported for participants in the elamipretide (n = 5/109 [4.6%]) and the placebo groups (n = 3/109 [2.8%]) and were not deemed to be treatment related. The incidence of AEs leading to discontinuation was greater in the elamipretide group (n = 8/109 [7.3%] and n = 2/109 [1.8%] for placebo, respectively). No participants had an AE with an outcome of death or hospitalization.

**Table 4:**
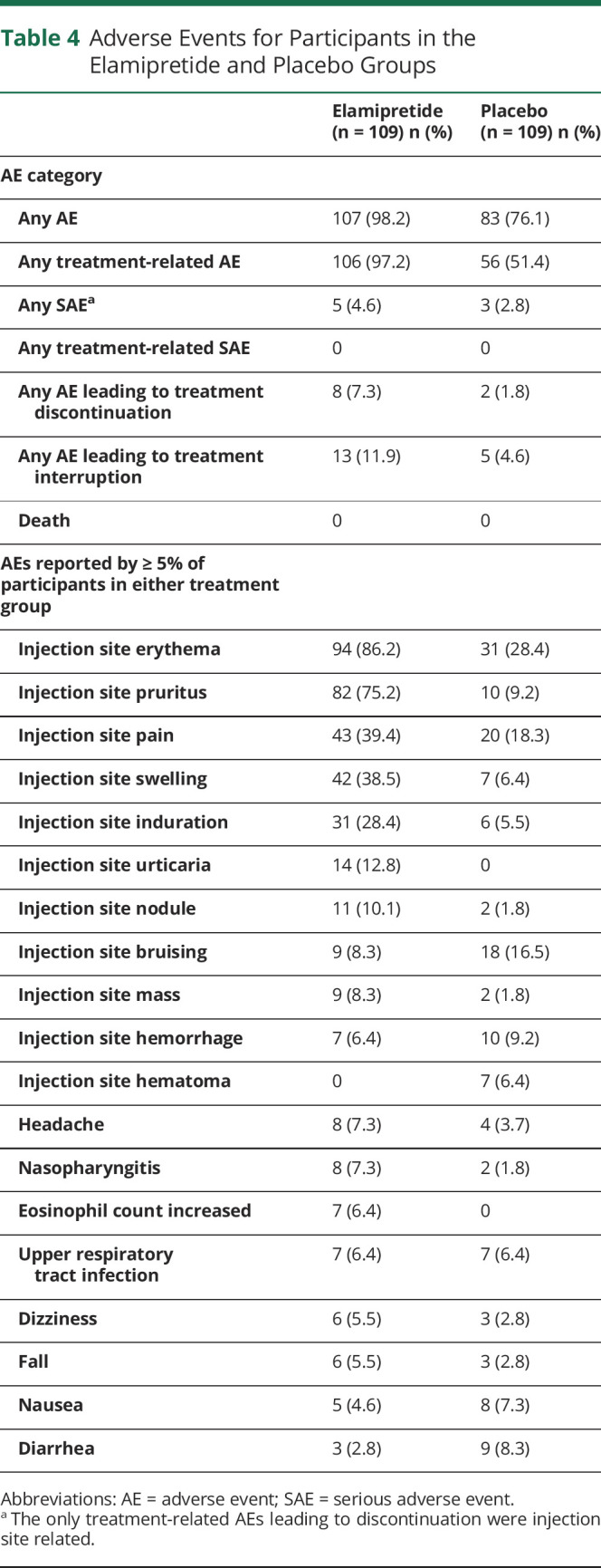
Adverse Events for Participants in the Elamipretide and Placebo Groups

### Pharmacokinetics

Population pharmacokinetic models were fit successfully to 3 analytes, elamipretide and 2 metabolites, M1 and M2. For elamipretide, systemic parameters scaled allometrically. No covariates influenced the systemic or absorption parameters. For M1 and M2, apparent clearance decreased with age and increased with renal function. No other covariates influenced the systemic parameters. In the exposure-response analysis, participants with an nDNA alteration had an increase in the change and fractional change at week 24 compared with that at day 1 (i.e., baseline) value for the 6MWT as a function of the elamipretide steady state area under the curve (*p* = 0.0262 and *p* = 0.0345, respectively).

## Discussion

We present the results of the first phase 3 trial in PMM with elamipretide. Overall, participants who received elamipretide did not meet either primary or secondary endpoints. Specifically, there were no statistically significant changes between elamipretide and placebo in the 6MWT or the PMMSA Total Fatigue Score. MMPOWER-3 uncovered several findings, specifically the importance of considering pathogenic genotypes within the PMM population when evaluating the effect of investigational treatments. Among the most interesting findings of the trial, identified in a post hoc analysis, was that participants with PMM with nDNA defects performed significantly better on the 6MWT, whereas participants with mtDNA alteration did not differ from placebo. The insight from these subgroups are novel findings and are expected to contribute substantially to future PMM studies.

Patient improvements in 6MWT and PMMSA TFS from this study showed a similar trend as those results obtained from the phase 1/2 (MMPOWER-1) and phase 2 (MMPOWER-2) clinical trials of elamipretide in patients with PMM.^10,11^ MMPOWER-1^10^ informed the dose selection for the phase 2 and 3 studies, while the results from the phase 2 study, MMPOWER-2,^11^ provided an efficacy signal and data to support the initiation of this phase 3 study.

While this study did not meet either of its primary endpoints (changes in the 6MWT and PMMSA Total Fatigue Score), participants treated with elamipretide did report slightly less total fatigue (between-group difference was not statistically significant) at the end of treatment, as assessed by the PMMSA Total Fatigue Score. Future studies are needed to elucidate whether the slight change in PMMSA Total fatigue score in treated and untreated participants is within the test variability range or a true measure of fatigue improvement not reaching statistical significance due to the mild-to-moderate impairment of participants at baseline and increased heterogeneity in participant selection.

The response in 6MWT in the nDNA cohort, as a function of plasma area under the curve (AUC0-24), demonstrates a statistically significant correlation, which supports this subgroup finding, and suggests that the therapeutic dose may not be optimized. It is possible that the exposure-response relationship may differ by genotype/phenotype. These findings warrant further investigation and clearly underscore the importance of considering genetic subtypes in mitochondrial myopathy and the drug mechanism of action. All the genes responsible for mtDNA maintenance are expressed in the nuclear genome.^33^ Mitochondrial proteins/enzymes that are synthesized from nDNA must be transported across the inner mitochondrial membrane, enriched with cardiolipin.^34^ These metabolite and nucleotide transporters depend on cardiolipin for their assembly and activity,^35,36^ and cardiolipin is known to stabilize mtDNA packaging into nucleoids.^37^ It is intriguing to speculate that elamipretide's benefit in the nDNA cohort was caused by improved enzyme/metabolite transport into mitochondria, improved assembly and morphology of mitochondria, augmented mtDNA stability, reductions in ROS, or any combination thereof. Although these presumptions are supported by preclinical work where elamipretide improved mitochondrial protein import and mitochondrial morphology,^13,27,38^ further investigation will advance our understanding of the 6MWT increase in the nDNA cohort.

Although the patient population with PMM included in the MMPOWER-3 study was impaired on the 6MWT at baseline compared with literature-based healthy controls (655 [±91] meters),^33^ the phase 3 population was only moderately impaired based on the average PMMSA fatigue score and only slightly more impaired than the population norm on the Neuro-QoL Fatigue Short-Form T score (mean T score ≥50 points at baseline). In these mildly to moderately impaired participants, elamipretide did not demonstrate statistically significant changes on the primary endpoints (6MWT and PMMSA TFS) from baseline to 24 weeks compared with placebo. Additional analyses involving functional and patient reported outcomes are necessary to assess the ability of elamipretide to affect positive changes in genetically defined subgroups of this patient population.

In this phase-3 study, elamipretide was generally well tolerated. Most AEs were mild to moderate in severity, with the most commonly reported AEs including injection site reactions. This safety was similar to that observed in the MMPOWER-2 study^11^ and the TAZPOWER study in patients with Barth syndrome with no serious AEs or deaths.^12^

There are important lessons to be learned from this clinical trial regarding trial design in PMD. The first is that a better understanding of the natural history of PMM will help in future studies. Although the RePOWER pretrial, noninterventional registry^30^ did not facilitate the ability to study effort-dependent endpoints, it did enhance the understanding of disease mechanisms and derisk/homogenize disease group selection for trial participants according to the specific drug-intended targets but not the effort-dependent endpoints.

The second lesson is that there is a clear need to further study meaningful endpoints in this patient population. Fatigue has been identified as the primary issue which this patient population experiences, identifying a definitive focal point to be addressed in future therapeutic trials. Refining the sensitivity of the PMMSA fatigue scores in PMD and PMM further to capture signals is tantamount for future studies to address data gaps.

The lack of available biomarkers for PMM also presented challenges. The variability of participant responses to the PMMSA Total Fatigue score, which is susceptible to a placebo effect were all challenges that skewed the objectivity of the study endpoints. Furthermore, the mild-to-moderate fatigue scores observed at baseline point to a lack of sensitivity of this endpoint. The identification of objective endpoints or biomarkers assessing PMM would be beneficial for future trial design because the availability of biomarkers helps to target individuals who are most likely to respond to treatment, providing the ability to verify target engagement, which could allow the use of enrichment strategies and reduce reliance on effort-dependent endpoints. For example, altered plasma acylcarnitine levels have previously been seen in patients with PMM,^39^ and elamipretide has been shown to reduce plasma acylcarnitines in other PMD,^40^ but the relation of this biomarker to clinically meaningful changes in daily life needs to be established.

The third lesson is from the basket design from MMPOWER-3, which pooled both nDNA and mtDNA participants. The placebo effect on 6MWT was prominent in mtDNA participants in the prespecified subgroup analysis, and given the size of this subgroup, this drove the placebo effect observed in the trial. Conversely, there was not a discernible placebo response on 6MWT in nDNA participants (depicted in Figure 3B), as identified in a post hoc analysis. Based on these observations, it seems that the MMPOWER-3 basket trial design introduced insurmountable heterogeneity. It has been shown that substantial efficiencies are possible in basket trial design only if the investigational drug works in most or all baskets in the clinical study, with losses of power and statistical significance if the investigational drug only works in a single basket.^41^ The basket trial design used in MMPOWER-3 introduced significant heterogeneity between participants with mtDNA and nDNA alteration, which was particularly apparent with 6MWT results seen in the prespecified subgroup analysis by genetic abnormality. Therefore, it is doubtful that a separation in efficacy between active treatment and placebo would have been observed regardless of the length of the trial. This critical lesson advances our understanding of the various PMM genotypes and highlights the need to critically consider particular genotypes in the design of future trials.

The MMPOWER clinical development program was the most advanced and complete in PMM and provided significant lessons regarding study design and patient enrollment parameters. MMPOWER-3 was the first trial that progressed into phase 3 to assess a therapy for participants with PMM. Although the primary endpoints were not met for the overall population, the observation of the improvement in the 6MWT in the nDNA subgroup is encouraging and hypothesis generating. Efforts are currently underway to confirm this positive benefit and these findings in a follow-up and targeted phase 3 trial in participants with PMM with pathogenic nDNA variants.

## Glossary

6MWT: 6-minute walk test
AE: adverse event
CGI: clinician global impression
eGFR: estimated glomerular filtration rate
ITT: intention to treat
LHON: Leber hereditary optic neuropathy
MMRM: mixed-model repeated measures
mtDNA: mitochondrial DNA
nDNA: nuclear DNA
PGI: patient global impression
PMDs: primary mitochondrial diseases
PMMs: primary mitochondrial myopathies
PMMSA: Primary Mitochondrial Myopathy Symptom Assessment
PP: per-protocol
PROs: patient-reported outcomes
QoL: quality of life
ROS: reactive oxygen species
TFS: total fatigue score
